# Supplementary material for: A quadratic model captures the human V1 response to variations in chromatic direction and contrast
Source: eLife. 2021 Aug 3;10:e65590. doi: 10.7554/eLife.65590 (PMC8452309; doi:10.7554/eLife.65590)
Supplement: Transparent reporting form [file elife-65590-transrepform.docx]

***eLife’s* transparent reporting form**

We encourage authors to provide detailed information *within their submission* to facilitate the interpretation and replication of experiments. Authors can upload supporting documentation to indicate the use of appropriate reporting guidelines for health-related research (see [EQUATOR Network](http://www.equator-network.org/%20)), life science research (see the [BioSharing Information Resource](https://biosharing.org/)), or the [ARRIVE guidelines](http://www.plosbiology.org/article/info:doi/10.1371/journal.pbio.1000412) for reporting work involving animal research. Where applicable, authors should refer to any relevant reporting standards documents in this form.

If you have any questions, please consult our Journal Policies and/or contact us: [editorial@elifesciences.org](mailto:editorial@elifesciences.org).

**Sample-size estimation**

- You should state whether an appropriate sample size was computed when the study was being designed
- You should state the statistical method of sample size computation and any required assumptions
- If no explicit power analysis was used, you should describe how you decided what sample (replicate) size (number) to use

Please outline where this information can be found within the submission (e.g., sections or figure legends), or explain why this information doesn’t apply to your submission:

A sample size of 3 was used in the experiment. This sample size was determined in advance, to be comparable in the total number of fMRI scanning hours (12 hrs) to other studies of cortical color processing. The number of subjects was pre-registered prior to data collection (OSF, links in Methods section).

**Replicates**

- You should report how often each experiment was performed
- You should include a definition of biological versus technical replication
- The data obtained should be provided and sufficient information should be provided to indicate the number of independent biological and/or technical replicates
- If you encountered any outliers, you should describe how these were handled
- Criteria for exclusion/inclusion of data should be clearly stated
- High-throughput sequence data should be uploaded before submission, with a private link for reviewers provided (these are available from both GEO and ArrayExpress)

Please outline where this information can be found within the submission (e.g., sections or figure legends), or explain why this information doesn’t apply to your submission:

Our experiment was performed two times for all subjects constituting a technical replication of the experiment. The replication was pre-registered after the initial data collection. Our exclusion/inclusion criteria were preregistered with OSF (links in Methods section) and are described in Methods. One subject moved while during the MRI scan creating an outlier relative the acceptable subject motion. The timepoints were removed via a standard procedure as explained in Methods.

**Statistical reporting**

- Statistical analysis methods should be described and justified
- Raw data should be presented in figures whenever informative to do so (typically when N per group is less than 10)
- For each experiment, you should identify the statistical tests used, exact values of N, definitions of center, methods of multiple test correction, and dispersion and precision measures (e.g., mean, median, SD, SEM, confidence intervals; and, for the major substantive results, a measure of effect size (e.g., Pearson's r, Cohen's d)
- Report exact p-values wherever possible alongside the summary statistics and 95% confidence intervals. These should be reported for all key questions and not only when the p-value is less than 0.05.

Please outline where this information can be found within the submission (e.g., sections or figure legends), or explain why this information doesn’t apply to your submission:

Data are analyzed and shown for each individual subject and replication. Both a general linear and a quadratic non-linear model were used to evaluate cortical responses to color the goodness of fits (R^2) were compared. Example fits of the model to the preproceesed fMRI time course data for each subject are shown in Figure 3 and corresponding supplemental figures. To assess if the model fits were different within and across subjects, we used cross-validation model comparison. To determine if the individual parameters of the model varied within and across subjects, a bootstrap analysis was used to generate 68% confidence intervals. The implentatation is described in Methods and in the Model appendix. The R^2 values for fits and confidence intervals are provided in Results.

(For large datasets, or papers with a very large number of statistical tests, you may upload a single table file with tests, Ns, etc., with reference to sections in the manuscript.)

**Group allocation**

- Indicate how samples were allocated into experimental groups (in the case of clinical studies, please specify allocation to treatment method); if randomization was used, please also state if restricted randomization was applied
- Indicate if masking was used during group allocation, data collection and/or data analysis

Please outline where this information can be found within the submission (e.g., sections or figure legends), or explain why this information doesn’t apply to your submission:

Our data analysis was performed on a single subject basis and thus there are no group allocations.

**Additional data files (“source data”)**

- We encourage you to upload relevant additional data files, such as numerical data that are represented as a graph in a figure, or as a summary table
- Where provided, these should be in the most useful format, and they can be uploaded as “Source data” files linked to a main figure or table
- Include model definition files including the full list of parameters used
- Include code used for data analysis (e.g., R, MatLab)
- Avoid stating that data files are “available upon request”

Please indicate the figures or tables for which source data files have been provided:

All MATLAB code used to analyze the data and generate the figures is available on our public GitHub code repository, as referenced in the paper.
